# Meeting Report on the Symposium “Evolutionary Applications” at the 3rd Joint Congress on Evolutionary Biology

**DOI:** 10.1111/eva.70082

**Published:** 2025-03-25

**Authors:** Hildegard Uecker

**Affiliations:** ^1^ Research Group Stochastic Evolutionary Dynamics, Department of Theoretical Biology Max Planck Institute for Evolutionary Biology Plön Germany

**Keywords:** conservation biology, domestication, drug resistance, gene drive, pesticide resistance

## Abstract

The symposium “Evolutionary Applications” took place on June 28, 2024 in the virtual part of the 3rd Joint Congress on Evolutionary Biology. It was contributed to the conference by the European Society for Evolutionary Biology (ESEB). The symposium highlighted research on evolutionary biology applied to address questions and contemporary problems in medicine and public health, conservation biology, and food production and agriculture. Each of the six talks covered a different application and a different organism: domestication of cheese‐making fungi, restoration of long‐lived bird populations, evolution of herbicide resistance, coral reef conservation, gene drive systems targeting Malaria vectors, and antibiotic resistance evolution in bacteria. By including speakers who are active in a consortium or work in an NGO, the symposium also showed how to make the step from scientific findings to practical application. The symposium furthermore featured a range of scientific methods, ranging from genomic analyses and mathematical modeling to laboratory evolution and field experiments. Speakers from across 15 time zones highlighted the potential of virtual symposia to foster global collaboration in evolutionary biology.

## 1 Introduction

Anthropogenic influence on the evolution of species—intentional or inadvertent—is ubiquitous, often with direct consequences for ecosystems, agriculture, and human health (Hendry et al. 2017; Baltazar‐Soares et al. 2021). This raises questions in evolutionary biology that are of immediate applied relevance. The symposium “Evolutionary Applications” at the 3rd Joint Congress on Evolutionary Biology brought together six researchers who apply concepts of evolutionary biology to address questions appearing in medicine and public health, conservation biology, and agriculture and food production. As pointed out afterwards by one of the symposium speakers, the symposium covered a “perfect set of topics to capture student interest,” inspiring them to include an overview of the talks in a large introductory course. Recordings of the symposium are available in the Evolution Meetings YouTube channel at (Part1 and Part2).

This report provides an introduction to the topics of the talks, followed by a brief discussion of connections between fields of application. Short summaries of each talk in chronological order with more detail than in the main text can be found in Appendix A.

## 2 The Topics Covered in the Symposium

### 2.1 Domestication

With the domestication and subsequent selective breeding of species for agricultural or other purposes, humans intentionally and rather strongly direct evolutionary change. Even though less apparent than the domestication of animals and plants (Kantar et al. 2019), microorganisms have also been subject to domestication and are pivotal in food production, for example, for making bread, wine, or cheese (Dupont et al. 2017). The first talk of the symposium was given by Tatiana Giraud (CNRS) on the domestication of *cheese‐making Penicillium* fungi. In the course of the talk, by the example of *Penicillium*, several themes appeared that are typical for domestication processes in general. The first is, of course, the selection of traits that are important for the purpose. In the case of cheese, desired traits include aroma, color, and reduced toxin production, and Tatiana Giraud and her team indeed found that these (and other) traits differed between cheese and non‐cheese lineages (Ropars, Caron, et al. 2020; Ropars, Didiot, et al. 2020; Dumas et al. 2020; Caron et al. 2021; Ropars and Giraud 2022; Crequer et al. 2023). However, domestication (and breeding) does not only involve artificial selection of desirable traits, but also leads to adaptation to the rearing or culture conditions themselves and often entails undesired evolutionary consequences such as a loss of genetic diversity, and Tatiana Giraud presented examples from *Penicillium* for both as well (Ropars, Caron, et al. 2020; Ropars, Didiot, et al. 2020; Dumas et al. 2020; Ropars and Giraud 2022). As also pointed out in the talk, studying the domestication process is not only of applied relevance but also provides insights into general evolutionary questions. Indeed, Charles Darwin starts his opus “On the origin of species” with a consideration of the domestication of animals and plants (Darwin 1859). Tatiana Giraud highlighted that domestication of cheese‐making fungi can serve to study parallel adaptation to a similar niche and identified parallel evolution for relevant traits in different cheese‐making fungi (Ropars, Caron, et al. 2020; Ropars, Didiot, et al. 2020; Ropars and Giraud 2022; Bennetot et al. 2023).

### 2.2 Conservation Biology

Anthropogenic environmental change such as habitat destruction, pollution, or climate warming puts species at risk of extinction. While species often evolve in response to habitat alterations, adaptive evolution risks to be too slow to rescue threatened populations from extinction. As population sizes decline, inbreeding depression poses an additional risk to population persistence. Conservation biology can greatly draw from insights from evolutionary biology. The talks by Madhavi Colton (Audubon California) and Jia Zheng (Beijing Normal University) addressed two complementary topics—optimal management strategies to support adaptive evolution and prevent population extinction and the risk inbreeding poses to the recovery of small populations.

#### 2.2.1 Management Strategies to Support Adaptation

It has been advocated that conservation strategies should take the evolutionary potential of populations into account and that management actions should be designed to facilitate adaptation (e.g., Eizaguirre and Baltazar‐Soares 2014). This is of particular importance in the context of climate change, which can—unlike some other stressors—not be entirely removed at the relevant timescale but at best be mitigated. Coral bleaching due to rising sea temperatures and the subsequent death of coral reefs has become a symbol for the danger that climate change poses to biodiversity. However, corals possess several features that give some hope for successful adaptation to a warming climate. These include large population sizes and huge offspring numbers as well as hybridization between related species (Willis et al. 2006), scope for symbiont‐mediated adaptation (Berkelmans and Van Oppen 2006; Drury 2020), and genetic variance in heat tolerance (Dziedzic et al. 2019; Drury 2020). In particular, the geographic distribution spans a variety of temperatures to which corals are locally adapted (Dixon et al. 2015). Madhavi Colton worked for almost 10 years at the Coral Reef Alliance—including as the Executive Director—which is a global NGO focusing on the protection of coral reefs (https://coral.org). In her talk, she presented results from eco‐evolutionary modeling that she and her collaborators performed to assess the adaptive capacity of corals and to identify the most effective conservation approaches for supporting the survival of coral reefs (Walsworth et al. 2019; McManus et al. 2021; Forrest et al. 2024). For coral reefs, conservation actions include the establishment of marine protected areas where local stressors, such as poor water quality or overfishing, are mitigated. For example, reduced fishing pressure can lead to the recovery of fish species that feed on macro‐algae which compete with corals for space. The findings from the modeling indicate that it is best to protect a diversity of reefs that are connected by dispersal—cold reefs as refuges from climate change and warm reefs as suppliers of pre‐adapted larvae dispersing into cold (but warming) reefs (Walsworth et al. 2019; Forrest et al. 2024). Yet, a reduction in carbon emissions remains key (Colton et al. 2022).

#### 2.2.2 Restoration of Populations From Low Numbers and Inbreeding

Habitat destruction, hunting, and other anthropogenic (and non‐anthropogenic) influences have driven many species to low numbers locally or globally. An important area of conservation biology concerns the recovery of small populations to sustainable sizes through appropriate conservation measures such as habitat restoration or protection from hunting. Translocation of individuals from one location to another or release of captive‐bred individuals can reintroduce species in areas where they were historically present but went extinct (Armstrong and Seddon 2008; Seddon et al. 2007), with numbers of founders often being low (Godefroid et al. 2011; Jamieson 2011). Yet, small populations—even though not all of them (Robinson et al. 2022)—are prone to suffer from inbreeding depression (Keller and Waller 2002; Huisman et al. 2016; Bozzuto et al. 2019; Armstrong et al. 2021). This may involve an increased risk of extinction (Keller et al. 1994; Newman and Pilson 1997; Nieminen et al. 2001) and—as pointed out by Jia Zheng—pose a threat to population recovery (Kardos et al. 2023). Jia Zheng's talk was driven by the question of how inbreeding depression and life‐history traits interact to shape restoration success in long‐lived birds with small founder populations (Zheng et al. 2024). In an individual‐based model, she and her collaborators found that inbreeding depression is expected to have a strong negative effect on population recovery in some species, but not in others, depending on their life‐history traits. Applied to crested ibis ( *Nipponia nippon* ), which thanks to conservation efforts recovered from only seven remaining individuals, the model accurately predicted the empirically observed population growth and inbreeding coefficient. Another key question is how to effectively expand a species' geographic range through multiple reintroductions. Jia Zheng compared two commonly used strategies in animal conservation programs (e.g., Schaub et al. 2009; Burt et al. 2016; Grossen et al. 2018), providing a much‐needed theoretical assessment to identify the optimal approach.

### 2.3 Resistance Evolution

While slow evolutionary responses pose a threat to biodiversity under anthropogenic environmental change, rapid evolution creates challenges in medicine such as resistance to antimicrobial drugs and cancer chemotherapy (Perron et al. 2015). Likewise, in agriculture, the evolution of pesticide resistance impairs the fight against weeds, insects, and plant pathogens. The evolution of resistance is affected by many factors, some rooted in the biology of the organism, such as the mode of reproduction of the organism (Uecker 2017), and others in the treatment strategy, such as the drug or pesticide dose (Kouyos et al. 2014; Mikaberidze et al. 2017). Understanding the dynamics of resistance evolution and characterizing the properties of resistant populations is key to developing sustainable treatment strategies and, similar to the study of domestication, provides insights into questions in evolutionary biology more broadly. Herbicide and antibiotic resistance were the topics of the talks by Regina Baucom (University of Michigan) and Ayari Fuentes‐Hernandez (Center for Genomic Sciences, UNAM), respectively.

#### 2.3.1 Herbicide Resistance

The cost of resistance is one of the key factors not only for the evolution but also for the persistence of resistance. The central theme of Regina Baucom's talk was the characterization of costs of herbicide resistance in the common morning glory (*Ipomoea purpurea*), a noxious wide‐spread weed that is usually treated with glyphosate (Roundup). Irrespective of the organism, costs of resistance are most often measured as intrinsic fitness costs that manifest in the organism grown in monoculture in the absence of other species, for example, as lower rates of germination of plants, as shown by Regina Baucom in her talk (Van Etten et al. 2016). However, individuals are usually part of a community that includes other species. For weeds, the surrounding community typically includes other weed species, pollinators, pathogens, and herbivores (Baucom 2019; Iriart et al. 2021). Resistance may alter species interactions, which in turn may negatively affect the fitness of the focal species, for example, herbicide resistance in weeds can increase damage through herbivory (Gassmann and Futuyma 2005). Such costs are termed ecological costs (Strauss et al. 1999) and are much less studied than intrinsic costs. In a large field experiment, Regina Baucom and her team discovered that resistance provided an ecological benefit instead of an ecological cost. In their experiment, glyphosate use increased insect herbivory of sensitive weed plants; herbicide‐resistant plants were in turn less damaged by herbivory than sensitive plants in the presence of glyphosate, for which Regina Baucom has tentative explanations based on the mechanism of glyphosate action (Zhang and Baucom 2025). This shows that the alteration of species interactions through resistance evolution may increase, rather than decrease, the fitness of the focal species, and that it may show an effect in the presence rather than in the absence of pesticides.

#### 2.3.2 Antibiotic Resistance

Chromosomal resistance to antibiotics (or other drugs or pesticides) is usually conferred by a spectrum of mutations that differ in their strengths of resistance, their costs in the absence of antibiotics, and the frequency at which they spontaneously appear. Which of them emerge and rise to high frequencies and possibly accumulate in the same genome during antibiotic treatment—and how fast—depends, among other factors, on the selection pressure imposed by the drug(s), the evolutionary history of the population, and chance (Peña‐Miller et al. 2013; Harmand et al. 2017, 2018; Santos‐Lopez et al. 2021); compensatory mutations might eventually alleviate costs (Maisnier‐Patin and Andersson 2004). In her talk, Ayari Fuentes‐Hernandez presented results from experimental evolution in *Escherichia coli* and mathematical modeling showing that resistance evolves more rapidly when selection is strong, but resistance mutations are more stably maintained in the absence of drug if selection was mild (Cisneros‐Mayoral et al. 2022). Resistance was achieved by different mutations in the two selection regimes. During a subsequent drug‐free period, populations became less resistant over time, but populations that had evolved during mild selection better maintained resistance mutations throughout. When antibiotic pressure was resumed, this allowed them to regain high levels of resistance as fast as populations that were both times exposed to strong selection, highlighting the role of the evolutionary history in resistance evolution. Besides the speed of resistance evolution and the stability of resistance mutations, it is also highly relevant whether resistance to one antibiotic turns cells simultaneously more or less susceptible to another antibiotic, known as collateral sensitivity or cross resistance. Ayari Fuentes‐Hernandez briefly addressed this topic at the end of her talk, which is, for example, of high relevance for the design of combination or sequential therapies (Pál et al. 2015; Aulin et al. 2021; Roemhild and Andersson 2021).

### 2.4 Genetic Biocontrol

Genetic biocontrol consists of the release of genetically modified organisms to control invasive species, pests, or disease vectors. A classic approach, which has already been successfully applied, is the sterile‐male technique, in which a population is inundated by sterile males (e.g., Vreysen et al. 2000). Yet, since sterile males only persist for one generation, it is restricted to the control of small populations. By contrast, gene drive systems with a higher than Mendelian inheritance can spread through the target population and can be designed to either suppress the population through strongly harmful effects or to replace it with a harmless genotype, for example, a mosquito that does not transmit disease. Such systems have been proposed by Kidwell and Ribeiro (1992) and Burt (2003) and have become a real possibility due to modern synthetic biology such as the CRISPR‐Cas technology. In his talk, Austin Burt (Imperial College London) provided an introduction to genetic biocontrol in general and specifically presented progress made in applying gene drive systems to control Malaria vectors in Africa. With respect to the latter, he first showed successful developments in research: the successful design of a gene drive in *Anopheles gambiae* —based on seminal theoretical work (Burt 2003) and tested in cage experiments (Kyrou et al. 2018)—and results from models predicting success in Malaria suppression upon release in West Africa (Hancock et al. 2024). He then also provided insights into other steps needed for eventual application: regulatory approval and stakeholder acceptance as well as the building of capacities needed for actual implementation (Burt et al. 2018). This includes, for example, a step‐by‐step progression, where field trials are first performed with non‐gene‐drive genetically modified mosquitoes, and outreach activities in African communities. The work presented in the talk was performed with the support of the non‐for‐profit consortium “Target Malaria” (www.targetmalaria.org), whose origins go back to an initiative by Austin Burt more than 20 years ago and that unites teams from Africa, North America, and Europe.

## 3 Connections Between the Fields of Application and Concluding Considerations

While the six talks addressed topics in disparate fields of application, the underlying evolutionary questions are often of interest in more than one field. For example, the general topic of domestication is not only of relevance in food production but also in conservation biology, such as in captive breeding programs with the subsequent release of animals into the wild for restoration or population augmentation (Frankham 2010; Schulte‐Hostedde and Mastromonaco 2015). While those programs have essentially the opposite goal of domestication, adaptation to the rearing conditions (or the production farm environment for plants) likely still occurs and may severely affect fitness in the wild (Araki et al. 2007; Williams and Hoffman 2009; Milot et al. 2013; Espeland et al. 2017; Pizza et al. 2021). Similarly, the rearing of insects for biocontrol may lead to animals with reduced fitness in the wild as they adapt to the cage environment, which could negatively affect control efforts (Leftwich et al. 2016). Domestication and conventional breeding have also led to crops that have lost resistance to pests because resistance often comes at a cost or is associated with bad taste (Karlsson Green et al. 2020), which indirectly links the fields of domestication and pesticide resistance research. Similar to the importance of domestication across fields, gene drive strategies cannot only be used to suppress disease vectors but also to control agricultural pests or invasive species, as Austin Burt also pointed out in the introduction of his talk (Wedell et al. 2019; Legros et al. 2021).

The different applied fields can mutually benefit by sharing concepts and insights among them. Similar to the evolution of resistance to drugs or pesticides, gene drive strategies are at risk of becoming ineffective due to resistance evolution (Price et al. 2020; Gomulkiewicz et al. 2021). It has been suggested that insights from antimicrobial or pesticide resistance research could be harvested to hamper resistance to gene drives (Burt 2003; Leftwich et al. 2016). For example, for CRISPR‐based gene drives, which cleave a target sequence to copy themselves in, multiple gRNAs can be stacked to target multiple sites in the target gene, increasing the genetic barrier to resistance, which follows the same rationale as antibiotic combination therapy (Khatri and Burt 2022). Furthermore, a better integration of research on resistance to drugs or pesticides in different organisms would be highly insightful, not only through identification of similarities but also of differences that appear through the specific biology of the organisms such as the modes of reproduction, dispersal patterns, and other aspects of the life cycle (Peck 2001; Rex Consortium 2007; Baucom et al. 2021). Lastly, insights from resistance evolution might be helpful in conservation biology and vice versa (e.g., Alexander et al. 2014; Gatenby et al. 2020). The field of evolutionary rescue forms an umbrella for research on adaptation to severe stress that, without adaptive evolution, would cause population extinction (Alexander et al. 2014; Carlson et al. 2014; Bell 2017).

The examples in the previous two paragraphs highlight the benefits of knowledge exchange across areas of application. An important takeaway from the symposium is, furthermore, the value of mathematical modeling in understanding biological processes, which was demonstrated in four of the six talks in different fields of application. Mathematical models can also help to generalize across fields by abstracting from specific organisms.

Finally, making the step from scientific research to real‐life impact involves convincing stakeholders, solving logistic problems, etc. This was impressively showcased in Austin Burt's talk, but of course applies to most or all areas of application. To contribute to bringing research to application, researchers may follow a career in an NGO (as Madhavi Colton does) or a government organization. However, as the example of Austin Burt shows, researchers within academia can also become active by establishing (or joining) interdisciplinary consortia that focus on bringing research to action.

Overall, the symposium highlighted the key role that evolutionary biology plays in solving problems central to biodiversity, food security, and human health. Evolutionary biologists can contribute to overcoming these problems by acquiring fundamental insights into the underlying biological systems, by applying evolutionary thinking to develop practical solutions, and by supporting the steps to actual implementation. The six talks featured these different kinds of contributions. Shared evolutionary principles call for cooperation across fields of application.

## Consent

All speakers have approved the publication of the meeting summary.

## Conflicts of Interest

The author declares no conflicts of interest.

## Data Availability

The author has nothing to report.

## Appendix A Summaries of the Six Talks in Chronological Order

### Domestication as a Model of Evolution: *Penicillium* Fungi Used for Making Cheese

In her talk, Tatiana Giraud (CNRS) presented both genomic and phenotypic comparisons between different species of cheese‐making and non‐cheese‐making *Penicillium* fungi. *P. camemberti* and *P. roqueforti* are genetically distant and have been domesticated independently, providing a model system to study adaptation to the same ecological niche and potential parallel adaptation. Traits of importance in the ecological niche of cheese include color, aroma, metabolism of sugar, proteins, and lipids, reduced toxin production, competitive exclusion of contaminants, and salt tolerance. Tatiana Giraud explained that recent horizontal transfer of “starship elements”, a type of mobile genetic element, has likely contributed to adaptation to this niche in several species of *Penicillium*. In the largest part of the talk, she provided a rich set of results from a comparison of different cheese‐making and non‐cheese‐making lineages of *P. roqueforti* with each other. Cheese‐making lineages indeed display adaptation in several of the adaptive traits, producing very little toxin, showing more blue color, and generating a better aroma than lineages from silage or lumber or food spoiling fungi. Similar observations hold for *P. camemberti*, which is used to produce Camembert cheese, in a comparison to the food spoiler *P. fuscoglaucum*. Tatiana Giraud also presented examples of fungi at an intermediate step of domestication, displaying intermediate phenotypes. While better adapted to the cheese niche, cheese‐making fungi produce few sexual spores, which likely is an evolutionary consequence of asexual propagation, and some cheese‐making lineages have little to no genetic diversity due to strong bottlenecks at domestication. Unlike *P. camemberti* (and the *P. roqueforti* lineage that is used for all blue cheeses other than Roquefort and Termignon cheese), the lineage of *P. roqueforti* that is used to make Roquefort has maintained genetic diversity, showing opposite effects of the product protection granted under the “Protected designation of origin” label.

### How Does Inbreeding Depression Influence the Restoration and Reintroduction of Long‐Lived Species?

Jia Zheng (Beijing Normal University) talked on population restoration (defined here as recovery from low population sizes) of long‐lived species. Empirical examples give a mixed picture with inbreeding depression preventing recovery in some but not in other species. Focusing specifically on long‐lived birds, Jia Zheng and collaborators developed an individual‐based model to test how stage‐structured mortality and fecundity influence whether inbreeding depression substantially lowers the success probability of population recovery after a bottleneck or not. The model predicts that, when mapping trait values of 41 species from the COMADRE data set onto the space of life‐history traits, inbreeding depression has little effect for some, but a very strong effect for others. Jia Zheng proceeded to considering the example of crested ibis ( *Nipponia nippon* ) in more detail. Predictions from the simulation model for the time of successful restoration and for the inbreeding coefficient are in remarkable agreement with the empirical data from a successful restoration, where an initial population of seven individuals grew to 9000 individuals within 40 years due to conservation efforts. Crested ibis is a residential species that is unlikely to sufficiently expand its geographic range on its own, which raises the question how reintroduction in multiple areas is best achieved. In the last part of the talk, Jia Zheng compared two strategies that are both used in animal reintroduction programs: picking the founders for each reintroduction at a new location from the same source population (“firework style”) or a reintroduction in a stepping‐stone manner, where a restored population provides the founders for the reintroduction attempt in the next location. The model showed that the firework style strategy is much more promising.

### Herbicide Adaptation as a Model for Integrating Across Genetics, Ecology, and Evolution

Regina Baucom (University of Michigan) presented her group's research about resistance of the common morning glory (*Ipomoea purpurea*) to glyphosate, which is the active compound in the widely‐used herbicide Roundup. A large greenhouse experiment with 10.000 plants from various locations across the United States demonstrated heterogeneity in the level of resistance with some highly resistant and some susceptible populations. Resistance is associated with lower rates of germination or a lower early growth rate, and resistant populations furthermore display higher rates of selfing. However, weeds do not grow in isolation but are part of a community consisting for example of other weeds, pollinators, diseases, and herbivores. One of the key questions of the talk was whether resistance also carries an ecological cost, that is, whether resistance alters interactions with other members of the community, and if so, whether these altered interactions affect the fitness of *Ipomoea purpurea*. Specifically, Regina Baucom's team assessed the relationship between herbicide resistance and resistance to insect herbivory, measuring damage due to herbicide treatment and to herbivory in a common garden experiment. Herbivore damage was greater in treated than untreated populations. If treated, plants resistant to glyphosate also displayed greater resistance to herbivory. Genomic analyses previously performed in the lab had found that glyphosate resistance is caused by detoxification or altered translocation of compounds, which may restore pathways that are simultaneously important for herbivore defense. Overall, the results demonstrate that instead of an ecological cost, herbicide resistance is correlated with herbivory resistance in the common morning glory in the presence of herbicides.

#### Additional References

Kuester, A. , Chang, S. M. , Baucom, R. S. (2015). The Geographic Mosaic of Herbicide Resistance Evolution in the Common Morning Glory, *Ipomoea purpurea* : Evidence for Resistance Hotspots and Low Genetic Differentiation Across the Landscape. Evolutionary Applications, 8(8), 821–833.26366199 10.1111/eva.12290PMC4561571

Kuester, A. , Fall, E. , Chang, S.M. , Baucom, R. (2017). Shifts in Outcrossing Rates and Changes to Floral Traits Are Associated With the Evolution of Herbicide Resistance in the Common Morning Glory. Ecology Letters, 20:(1), 41 *–* 49.27905176 10.1111/ele.12703

Gupta, S. , Harkess, A. , Soble, A. , Van Etten, M. , Leebens‐Mack, J. , Baucom, R.S. (2023). Interchromosomal Linkage Disequilibrium and Linked Fitness Cost Loci Associated With Selection for Herbicide Resistance. New Phytologist, 238:(3), 1263–1277.36721257 10.1111/nph.18782

### An Eco‐Evolutionary Approach to Coral Conservation

Madhavi Colton (Audubon California) shared insights from her research at the Coral Reef Alliance, where she had worked in various roles, including as the Executive Director, for almost 10 years. Coral reefs are threatened by local stressors such as overfishing or exposure to wastewater as well as by climate change. Warming temperatures make the animal host expel the symbiotic algae that perform photosynthesis and provide their host with resources, which leads to coral bleaching and ultimately starvation of the corals. Madhavi Colton's talk turned around the question whether corals can adapt to climate change and which conservation actions best support them in doing so. Projections of future coral reef cover from a series of modeling studies—some of them based on real‐word dispersal data between reefs—not only demonstrated the importance of genetic variation and dispersal for successful adaptation but also highlighted that only protecting cold reefs as climate refugia is not the most promising strategy. Instead, reducing local stressors across a diversity of inter‐connected reefs is important, since hot reefs, while likely doomed themselves, act as sources of heat‐tolerant larvae that can promote adaptation in cold reefs subject to warming. The models furthermore clearly show the crucial importance of reducing emissions. Actions are thus needed at local scales (e.g., improvements of water quality), regional scales (e.g., design marine protected areas of inter‐connected reefs allowing for dispersal), and global scales (reduce emissions). During the questions following the talk, Madhavi Colton explained how NGOs such as the Coral Reef Alliance that are active in various geographical locations contribute to the implementation of these actions.

#### Additional Reference

McManus, L.C. , Tekwa, E.W. , Schindler, D.E. , Walsworth, T.E. , Colton, M.A. , Webster, M.M. , Essington, T.E. , Forrest, D.L. , Palumbi, S.R. , Mumby, P.J. , Pinsky, M.L. (2021). Evolution Reverses the Effect of Network Structure on Metapopulation Persistence. Ecology, 102:(7), e03381.33942289 10.1002/ecy.3381PMC8365706

### Prospects for Genetic Biocontrol

Austin Burt (Imperial College London) described successful steps made towards applying gene drive systems to control Malaria vectors (mosquitoes). Gene drive systems can be used to deliver harmful genes and spread rapidly since they are inherited at higher frequencies than by Mendelian inheritance. They are thus suitable to control large populations either by suppressing them (as in the talk) or by replacing them by a non‐hazardous genotype, for example, one that does not transmit Malaria. In his talk, Austin Burt focused on a gene drive system that is based on the homing reaction: In such a construct, a homing endonuclease gene (HEG) is incorporated into a target gene in the host chromosome, disrupting the gene's function. If present in a heterozygote individual, it cleaves the target gene on the other host chromosome, leading to repair with the HEG containing copy as a template and thus creating an HEG homozygote. If the construct is expressed in the germline, more than 50% of the gametes of the heterozygous individual will carry the gene drive allele (> 95% in the gene drive system presented in the talk). The HEG construct should not have a deleterious effect in the heterozygote (to allow its spread) but have a strongly negative effect in homozygotes (to control the population) and furthermore target a functionally important sequence in which mutations are likely to be highly deleterious (to reduce the risk of resistance). The gene drive system presented in the talk renders female homozygous mosquitoes sterile and indeed consistently led to population crashes in small and large cage experiments. Results from models, parameterized with genomic data to estimate population sizes and movement patterns, furthermore showed that releases across 16 areas in West Africa resulted in a 90% population suppression in most areas and substantial Malaria suppression, provided the right vector species was targeted. Nevertheless, besides technical success (which also includes to minimise the risk of resistance), the gene drive system also needs to pass regulatory approval and be accepted by the stakeholders, and the capacities for employment need to be built. In the last part of the talk, Austin Burt described how he and others address those requirements, performing first trials with non‐gene‐drive genetically modified mosquitoes, contributing to information material at the global scale (e.g., by the WHO), and engaging the public at the local scale by open days in insectaries in London and in Africa and by other activities in African communities. Before concluding his talk, he briefly pointed to other gene drive systems that persist without spreading, which may be desirable for other applications. The research and activities presented in the talk were conducted under the auspices of the non‐for‐profit research consortium “Target Malaria”, which unites teams from Africa, North America, and Europe.

#### Additional References

Burt, A. & Deredec, A. (2018). Self‐Limiting Population Genetic Control With Sex‐Linked Genome Editors. Proceedings of the Royal Society of London B, 285, 1–9.10.1098/rspb.2018.0776PMC608325730051868

Connolly, J.B. , Mumford, J.D. , Fuchs, S. , Turner, G. , Beech, C. , North, A.R. , Burt, A. (2021). Systematic Identification of Plausible Pathways to Potential Harm via Problem Formulation for Investigational Releases of a Population Suppression Gene Drive to Control the Human Malaria Vector *Anopheles gambiae* in West Africa. Malaria Journal 20, 1–69.33781254 10.1186/s12936-021-03674-6PMC8006393

Willis, K. & Burt, A. (2024). Engineering Drive‐Selection Balance for Localised Population Suppression With Neutral Dynamics. BioRxiv 2024.05.21.595228 10.1101/2024.05.21.595228.PMC1183120739903106

### The Role of Selection and Evolutionary History in Microbial Evolution of Antibiotic Resistance

Ayari Fuentes‐Hernandez (Center for Genomic Sciences, UNAM) combined experimental evolution in *Escherichia coli* and mathematical modeling to assess the role of selection and evolutionary history in antibiotic resistance evolution. In the first phase, bacterial populations were subject to a so‐called adaptive ramp, where during each growth phase the concentration of the antibiotic—the beta‐lactam Ampicillin in the experiment—is chosen to maintain a certain level of growth inhibition; this requires increasing concentrations over time as the population keeps evolving. Populations were selected for higher resistance, either applying mild or strong selection pressure, until the minimal inhibitory concentration (MIC) had increased 10‐fold. In the second phase, the same populations grew in a drug‐free environment, followed by another ramp selection in phase 3. The model assumed the presence of two resistance alleles: one mutation confers low resistance entailing a low cost in the absence of drug; the other one confers strong resistance, however at a high cost in a drug‐free environment. The model and the experiment agree in key results: (1) Adaptation in phase 1 is faster with strong than with mild selection. (2) The level of resistance decreases in phase 2; resistance mutations suffer more loss in phase 2 if populations had been subject to strong selection than if they had been subject to mild selection in phase 1. (3) Adaptation in phase 3 is rapid, irrespective of the selection pressure. Sequencing of the experimentally evolved populations showed that the same mutations are mostly maintained across all three phases if the selection pressure in phase 1 and 3 is mild. By contrast, with strong selection, mutations acquired in phase 1 were lost in phase 2, and different mutations spread in phase 3. Key insights of the research are that the strength of selection shapes the set of mutations that spread and that mild selection leads to more stable resistance mutations. At the end of the talk, Ayari Fuentes‐Hernandez presented preliminary data about cross resistance or sensitivity of the evolved populations to other antibiotics, showing cross sensitivity with beta‐lactams for both selection pressures.
